# Sap Flow Velocity in *Fraxinus pennsylvanica* in Response to Water Stress and Microclimatic Variables

**DOI:** 10.3389/fpls.2022.884526

**Published:** 2022-05-10

**Authors:** Yu Su, Xinyu Wang, Yiqiu Sun, Hailong Wu

**Affiliations:** College of Environmental Science and Security Engineering, Tianjin University of Technology, Tianjin, China

**Keywords:** sap flow, semiarid regions, *Fraxinus pennsylvanica*, driving factors, partial correlation

## Abstract

In arid and semiarid regions with water shortage, forestry development is limited by water availability. Understanding how tree sap flow responds to water stress and microclimatic variables is essential for the management of trees and the understanding of the eco-physiological properties of trees in arid areas. In the city of Tianjin in northern China, we measured the sap flow of *Fraxinus pennsylvanica*, a widely distributed urban greening tree species in semiarid regions of China. We measured the sap flow in four *F. pennsylvanica* trees over 6 months (April–September 2021), using a thermal diffusion probe method, and simultaneously monitored microclimatic variables and soil moisture. Results indicated that high nighttime sap flow velocity might be produced under the water stress condition. In addition, the nighttime sap flow velocity under the water stress condition was more susceptible to the combined effects of meteorological factors at night. The daytime sap flow velocity exerted a highly significant positive effect on the nighttime sap flow velocity during the whole research period, and the model fit was higher in the early growing season than that in the late growing season (early growing season: *R*^2^ = 0.51, *P* < 0.01; late growing season: *R*^2^ = 0.36, *P* < 0.01). Vapor pressure deficit had a positive effect on daytime sap flow. However, net vapor pressure deficit restrained daytime sap flow velocity when the intercorrelation between the microclimatic variables was removed. Our study highlights that drought areas perhaps have higher nighttime sap flow and that more emphasis should be placed on nighttime sap flow and the response of nighttime sap flow to microclimatic variables. In addition, the influence of other microclimatic variables on vapor pressure deficit needs to be considered when analyzing the relationship between daytime sap flow and vapor pressure deficit. An increase in net VPD can suppress the daytime sap flow.

## Introduction

Liquid water entering the root system from the soil is transported from the conducting tissue to leaves under transpiration pull. The sap flow along the conducting tissue of plants is indispensable for maintaining the hydraulic relationship between soil and atmosphere, supplying oxygen to xylem parenchymal cells, and facilitating nutrient uptake (Hubbart et al., 2007). Sap flow is an essential indicator of tree growth conditions, and water loss estimation, including dynamic changes and influencing factors, has been extensively investigated from the perspective of a single tree and stand (Paudel et al., 2015; Kupper et al., 2018). The traditional theory assumes that sap flow only occurs during the day because leaf stomata of trees and other C3 plants close at night. However, emerging research shows that nighttime sap flow is observed for many species and in a range of habitats (Yu et al., 2019; Fricke, 2020). Therefore, neglecting nighttime transpiration will lead to the underestimation of vegetation water consumption in the study of plant transpiration (O’Keefe et al., 2020). In addition, global climate observations demonstrated that land warms faster at night than during the day, and this trend can lead to an increase in plant water requirement at night (Zeppel et al., 2015). The nighttime sap flow movement in plants can facilitate the transport of nutrients and oxygen for plant growth, which in turn affects plant growth status and productivity (Chen et al., 2020b; Liu et al., 2021).

Furthermore, nighttime sap flow plays an important role in removing embolisms and enhancing the adaptation of plants to drought conditions (Fricke, 2019). Therefore, the nighttime sap flow is as significant as daytime sap flow in the study of tree transpiration. Previous studies on impact factors reported that vapor pressure deficit (VPD) and solar radiation (R_s_) are dominant factors affecting the sap flow during the day and VPD is the critical driving factor related to nighttime sap flow (Di et al., 2019). Although critical driving factors vary during the day and night, the nighttime sap flow is partly a supplement to the water deficit caused by transpiration during the day. Stomatal activity and physiological regulation of plants demonstrate a certain relationship despite their difference during the day and night (Kupper et al., 2018). Analyzing the relationship between daytime and nighttime sap flow as well as their similarities and differences are necessary. Therefore, we hypothesize that daytime and nighttime sap flow velocities are slightly correlated despite the difference among the dominant influencing factors.

The sap flow in the soil–plant–atmosphere continuum is mainly influenced by environmental factors and its own physiological control. Soil moisture is the main source of plant sap flow because it affects the overall level of sap flow (Zhao et al., 2017; Montoro et al., 2020). Previous studies indicated that R_s_ and VPD are the main factors that influence transpiration in a single tree in terms of meteorological factors affecting plant sap flow (Deng et al., 2021). Another study indicated that solar radiation is the maximum impact factor and potential evapotranspiration and solar radiation are positively correlated with relative changes in the sap flow volume of plants with an *R*^2^ of 0.75 (Sun et al., 2021). However, the degree of influence of meteorological factors on sap flow varies for different moisture conditions. A previous report indicated that drought weakens the extent to which meteorological factors can explain changes in tree water use (Di et al., 2019). Discussing the relationship between sap flow and meteorological factors by dividing soil moisture into periods of sufficiency and stress is necessary. We hypothesize that the response of *Fraxinus pennsylvanica* to meteorological factors varies under different moisture conditions.

The sap flow of urban greening tree species is affected by both global warming temperatures and intensification of the urban heat island effect under the current trend of climate change (Roetzer et al., 2021). The increased water requirements of urban trees and limitations of water resources are both issues that must be investigated and addressed. However, studies on daytime sap flow in trees have mainly been carried out in plantations. The lack of information and studies on the diurnal sap flow of urban landscape trees has led to the underestimation of plant water consumption and subsequent decline in their growth and longevity (Wu et al., 2020). Therefore, the common green tree species of *F. pennsylvanica* found in North China is used as the research object of this study to investigate diurnal sap flow change patterns. This work aims to provide a theoretical basis for water requirements of *F. pennsylvanica* and irrigation strategies for urban green spaces essential in the sustainable development and management of cities. We hypothesize that (1) the relationship between nighttime and daytime sap flow velocity of *F. pennsylvanica* is significantly correlated but responds differently to driving factors and (2) the response of sap flow velocity to meteorological factors also varies under diverse soil moisture conditions (with and without water stress). Sap flux velocity of *F. pennsylvanica* was monitored from April to September 2021 using a thermal diffusive probe system to test the hypotheses. The analysis mainly aimed to (1) identify the dynamic characteristics of daytime and nighttime sap flows, (2) ascertain the relationship between sap flows under different moisture conditions and meteorological factors, and (3) determine the relationships between daytime and nighttime sap flows and variations in their responses to driving factors.

## Materials and Methods

### Study Site

The study was conducted at Tianjin University of Technology (38°51e st°51e s 116°51e s 1°20e s 11Xiqing District, Tianjin. The low and flat area is located in the northeastern part of the North China Plain at an altitude of about 5 m above sea level. The region is characterized by a warm temperate semihumid continental monsoon climate with four distinct seasons (Chao, 2019). The mean annual air temperature is 11.6°C and the mean annual precipitation is 586.1 mm, including 443.2 mm in the summer. The average annual sunshine is 2810.4 h, the average annual relative humidity is 63%, and the average annual evaporation is 1709.7 mm (Tianjin Meteorological Bureau).

The tree species used in the experiment was *F. pennsylvanica*, which is a major greening species in northern China. *F. pennsylvanica* can reach a maximum height of 10 m, and its growing season is from April to November. This tree is excellent for urban greening because of its characteristics of cold tolerance, low humidity requirement, salinity tolerance, and drought tolerance (Aiyun et al., 2019). Four trees were selected and used as samples according to the conditions of the study site (Table 1). The straight and well-grown trees demonstrated no discernible effects of disease or insect damage. Tree diameter at breast height was measured using a breast diameter ruler, which was placed around the tree at the height of 1.3 m above the ground before directly reading the value. Tree height was measured with an ultrasonic altimeter (Vertex IV, Haglof Inc., Sweden) by maintaining the instrument in a vertical orientation, aiming the crosshairs at the measurement target, and directly reading the height of the tree. Crown width was measured using a measuring tape and compass. The average of all measurements was calculated three times in the east-west and north–south directions using tape.

**TABLE 1:** Basic parameters of sample tree.

Tree number	Height (m)	Stem diameter (cm)	Crown width (m)
1	9.30	15.60	6.17
2	6.90	10.20	4.68
3	10.40	27.20	8.73
4	8.10	16.70	7.35

### Sap Flow Measurements

We used the thermal diffusion probe (TDP) to monitor the *F. pennsylvanica* sap flow continuously between April and September 2021. A set of Granier-type thermal dissipation probes (TDP, Dynamax Inc., United States) was installed in each tree. Each set of thermal diffusion probes contains two probes: a reference probe containing a thermocouple inside it and a heating probe housing the thermocouple and heating element. The probes are 10 mm long and 2 mm in diameter. The heating probe is placed above the measuring position, and the reference probe is placed below the measuring position, with both probes placed parallel to the ground. The probes are inserted vertically at a height of approximately 1.3 m, with a spacing of 10 cm. The probes are installed on the north side of each sample tree at breast height (Jonard et al., 2011) because using the sap flow velocity on the north side of the tree generates a minimal error in estimating stand sap flow (Moon et al., 2017). Spherical foam was placed around the probes and then covered with aluminum foil to reduce the effects of solar radiation and prevent infiltration of rainwater. The sap flow velocity near the heating probes affects the measured temperature. On the basis of thermal diffusion theory of liquid rates, high sap flow velocity indicates that a large amount of heat is removed from the heating probe. A low heating probe temperature corresponds to a slight temperature difference between heating and reference probes. Therefore, the sap flow velocity can be calculated using the temperature difference between the upper and lower probes in the sample tree. A data collector (DT80, Data Taker Inc., China) was applied to determine the temperature difference between heating and reference probes every 10 s, and the average value was recorded every hour. The sap flow velocity (cm⋅h^–1^) was calculated from the differential temperature using the following empirical equation (Granier, 1987):

Ki=△⁢TM-△⁢Ti△⁢Ti, vi=119×10-4⁢Ki1.231×3600,

Where K_i_ is the parameter, ΔT_M_ is the maximum temperature difference between the upper and lower probes in 24 h (°C), ΔT_i_ is the instantaneous temperature difference value at a certain moment (°C), and V_i_ is the sap flow velocity (cm • h^–1^).

### Environmental Factor Measurements

Meteorological data, including atmospheric temperature (T_a_,°C), relative humidity (RH, %), solar radiation (R_s_, W⋅m^–2^), precipitation (P, mm), and other meteorological variables, were simultaneously measured with the sap flow through a small weather station (WatchDog 2000, SPECTRUM Inc., United States) at the study site. Vapor pressure deficit (VPD, kPa) was calculated from the measured T_*a*_ and RH using the following the empirical equation (Zhao et al., 2017):

$$\text{VPD}=\left(1-\text{RH}\right)\;\left(0.611\times e^{\frac{17.502\,{\text{T}}_{\text{a}}}{{\text{T}}_{\text{a}}+240.97}}\right).$$

A soil moisture monitoring system (HOBO, LI-COR Inc., United States) consisting of soil moisture sensors and a data collector was also installed in the experimental area to monitor the volumetric water content. The sensors are inserted vertically in the soil layer at depths of 5, 20, 30, and 40 cm around the root system of each sample tree (approximately 20 cm near the main roots and as close as possible to the roots without harming the root system). The soil volumetric water content is automatically recorded and the average value is obtained every hour and then stored in the data collector of the soil moisture monitoring system. A PC can be connected to read the soil moisture data directly. The soil relative extractable water (REW) was used to classify the soil moisture status and calculated as follows (Granier et al., 2000b; Chen et al., 2014b):

$$\text{REW}=\frac{\text{VWC}-{\text{VWC}}_{\text{min}}}{{\text{VWC}}_{\text{FC}}-{\text{VWC}}_{\text{min}}},$$

Where VWC is the volumetric soil water content (cm^3^ cm^–3^), VWC_FC_ is the soil water content at field capacity (VWC_FC_ was used max VWC during the study period due to unavailability of VWC at field capacity), and VWC_min_ is the minimum soil water content during the study period. The dimensionless REW is the relative effective water content of the soil that demonstrates values from 0 to 1. Plants are usually considered under soil water stress when REW < 0.4 (Chen et al., 2020b).

### Data Processing and Analysis

Although continuous monitoring was performed from April 2021 to September 2021, about 15% of data during the observation period were missing due to power outages and equipment failures. Microsoft Excel was used to convert the temperature difference data into sap flow data, and night was defined in hours when solar radiation was 0 W⋅m^–2^. The months of April, May, and June were designated as the early part of the *F. pennsylvanica* growing season and July, August, and September as the late part of the *F. pennsylvanica* growing season (Aiyun et al., 2019). Correlation data were processed with SPSS 22.0 using partial correlation and principal component analysis (PCA), and images were plotted using Origin 10.0.

## Results

### Variation in Daytime and Nighttime Sap Flow Velocity

**FIGURE 1:**
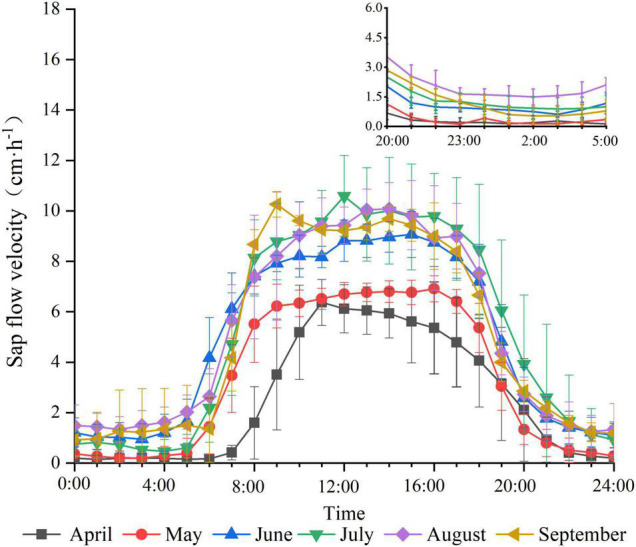
Daily variation of sap flow velocity by month.

The trend of sap flow velocity from April to September is shown in Figure 1. The daily variation of sap flow velocity in different months showed a single-peaked curve. The daytime sap flow velocity was significantly higher than the nighttime sap flow velocity. The sap flow velocity rises rapidly after starting early in the morning, reaches a peak, remains stable for a period of time, and finally decreases quickly until the evening. However, the magnitude of the sap flow velocity varies every month. The sap flow velocity in April and May is significantly lower and starts later than those in other months. The average daily sap flow velocity in April was the weakest during the study period at 1.609 cm⋅h^–1^ and the highest in July at 4.824 cm⋅h^–1^. Characteristics of nighttime sap flow velocity in different months (Figure 1) presented that the sap flow velocity is maintained at a flat and low level at night. The nighttime sap flow velocity is lower in April and May than that in other months. In addition, the sap flow is more active in the first half of the night than that in the second half of the night.

### Influencing Factors of Sap Flow Velocity

#### Characteristics of Soil Water Content

**FIGURE 2:**
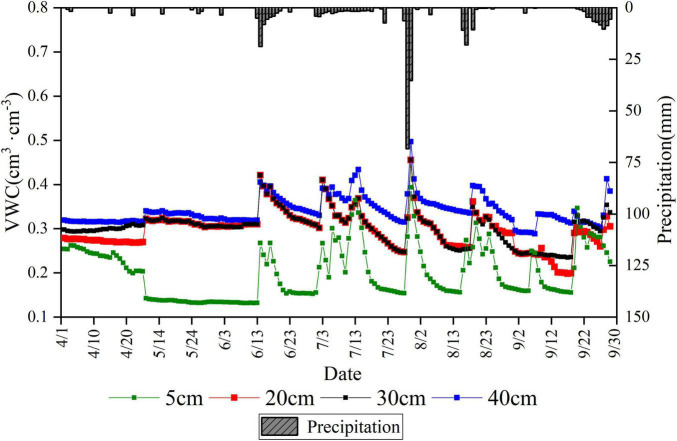
Variation of precipitation and daily average soil volumetric water content at the site.

The average daily soil water content of the soil layer with a depth of 40 cm was higher than that of 5, 20, and 30 cm. The moisture content of soil layers with a depth of 20 and 30 cm was similar (Figure 2). The dynamics of soil moisture all responded to rainfall events, especially continuous rainfall. For example, the average daily water content of soil layers with a depth of 5, 20, 30, and 40 cm increased from 0.154, 0.247, 0.248, and 0.314 cm^3^ cm^–3^ to 0.393, 0.456, 0.455, and 0.497 cm^3^ ⋅cm^–3^, respectively, for the largest continuous rainfall event in July (July 28–30) throughout the study period. The soil water content increased to some extent in all layers when continuous rainfall events occurred. Notably, the increase was high in the soil water content close to the surface layer. Soil moisture increased rapidly with each successive rainfall and decreased slowly during the subsequent consecutive rain-free days. However, differences were also observed in the magnitude of the decrease, with the surface soil moisture content decreasing rapidly. Large differences were also recorded in soil water content between months due to rainfall. Figure 2 and Supplementary Table 1 illustrated the satisfactory soil moisture conditions in July due to its many days of precipitation.

#### Characteristics of Microclimatic Factors and Response of Sap Flow Velocity to Meteorological Factors

**FIGURE 3:**
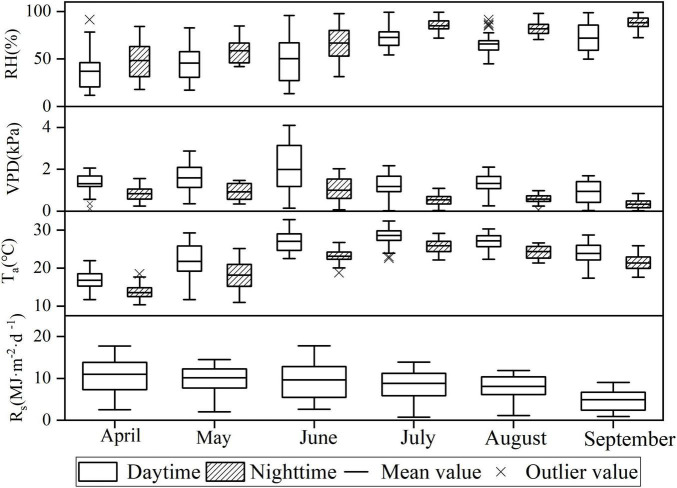
Variations of meteorological factors by month.

The box plot in Figure 3 clearly presented that the distribution of meteorological factor values differs significantly. The overall RH was higher at night than that during the day, while the temperature and water vapor pressure deficits were lower at night than those during the day. T_*a*_ and RH were higher, but R_s_ was not significantly higher in the summer months of June, July, and August compared with those in other months. Such finding is due to the intense rainy weather in summer that results in low average solar radiation. The sap flow velocity was highly significantly and positively correlated with both R_s_ and T_*a*_ for all months during the daytime, and the highest correlation was found between T_*a*_ and sap flow velocity in July with a correlation of 0.591. The sap flow velocity was significantly and positively correlated with T_*a*_ and VPD for all months at night. At night, the strongest correlation between sap flow velocity and microclimatic factor occurred in August, with a correlation of 0.489 between sap flow velocity and VPD (Table 2).

**TABLE 2:** Partial correlation between sap flow velocity and meteorological factors for each month.

Month	RH		T_a_		VPD		R_s_
	Daytime	Nighttime	Daytime	Nighttime	Daytime	Nighttime	Daytime
April	−0.057	0.230**	0.289**	0.169**	−0.063	0.267**	0.151**
May	−0.254**	0.231**	0.237**	0.148*	−0.128*	0.383**	0.462**
June	−0.441**	0.067	0.495**	0.161*	−0.420**	0.146**	0.294**
July	−0.108	0.268*	0.591**	0.432**	−0.118	0.256*	0.365**
August	−0.399**	0.438**	0.518**	0.295**	−0.336**	0.489**	0.315**
September	−0.395**	0.282	0.328**	0.409*	−0.322	0.176**	0.539**

**P < 0.01, *P < 0.05.

#### Response of Sap Flow Velocity to Microclimatic Factors Under Different Soil Water Content

The mode of action of the combination of factors on sap flow velocity under different circumstances must be considered, given that environmental factors exist interdependently when influencing sap flow. Soil moisture conditions can determine the overall level of tree transpiration (Deng et al., 2021). Hence, the sap flow velocity under different soil water conditions was analyzed according to meteorological factors of the same period. Sap flow and meteorological factor data of several days before and after typical rainfall during the study period were selected separately for analysis while considering the high degree of influence of rainfall on soil moisture. Available data before and after typical continuous rainfall events with and without water stress conditions in June and August were selected for the corresponding analysis. The comparison of sap flow velocity data corresponding to consecutive rainfall events demonstrated that the mean nighttime sap flow velocity is higher during all periods of water stress than those without water stress (Table 3). Under the water stress condition, the nighttime sap flow velocities in the two events were 1.79 ± 0.69 cm⋅h^–1^ and 1.91 ± 0.28 cm⋅h^–1^. Under the no-water stress condition, the nighttime sap flow velocities in the two events were 1.49 ± 0.21 cm⋅h^–1^ and 1.62 ± 0.26 cm⋅h^–1^. The correlation between sap flow velocity and meteorological factors also varied under different moisture conditions (Table 4). The results of the partial correlation analysis, excluding the correlation between environmental factors, indicated that sap flow velocity under water stress is only correlated with T_*a*_ during nighttime and the correlation coefficient was 0.357 (Table 4). However, Pearson’s correlation analysis, which takes into account interactions of environmental factors (Supplementary Table 2), shows that the correlation between sap flow and environmental factors was excellent. For example, nighttime sap flow velocity was highly significantly negatively correlated with RH under water stress with a correlation coefficient of 0.738, and highly significantly positively correlated with T_*a*_ and VPD with correlation coefficients of 0.458 and 0.696. In addition, the correlation between sap flow and environmental factors was generally higher at night with water stress than that without water stress. The combined effect of environmental factors was generally higher without water stress than that with water stress during the day (Supplementary Table 2). T_*a*_ was highly significantly and positively correlated with sap flow velocity regardless of day and night or soil water conditions (Table 4).

**TABLE 3:** Relationship between daytime and nighttime sap flow velocity under different water conditions.

Item	June rainfall event (June 14–21)		August rainfall event (August 16–20)	
	Water stress	No-water stress	Water stress	No-water stress
Daytime sap flow velocity (cm • h^–1^)	7.74 ± 0.27	8.13 ± 0.14	7.69 ± 0.27	8.04 ± 0.89
Nighttime sap flow velocity (cm • h^–1^)	1.79 ± 0.69	1.49 ± 0.21	1.91 ± 0.28	1.62 ± 0.26
Average soil water content (cm^3^ ⋅cm^–3^)	0.25 ± 0.01	0.33 ± 0.01	0.23 ± 0.01	0.31 ± 0.01
Total precipitation (mm)	50.29		40.38	

**TABLE 4:** Partial correlation between sap flow velocity and meteorological factors under different water conditions.

Water conditions	Daytime				Nighttime		
	RH	T_a_	VPD	R_s_	RH	T_a_	VPD
Water stress	−0.323**	0.441**	−0.460**	0.098	−0.103	0.357**	−0.102
No-water stress	−0.231**	0.313**	−0.118	0.454**	0.497**	0.321**	0.572**

**P < 0.01, *P < 0.05.

### Relationship Between Daytime and Nighttime Sap Flow Velocity

**FIGURE 4:**
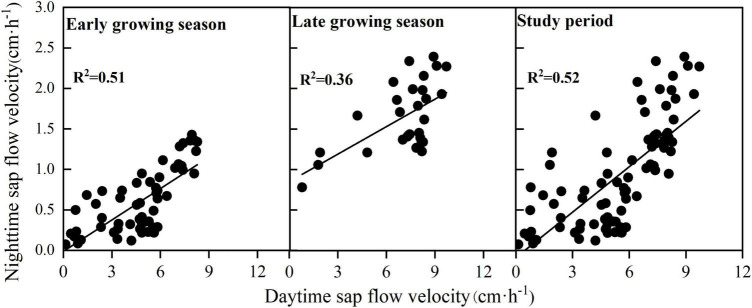
Relationship between daytime and nighttime sap flow velocity in different periods.

The nighttime sap flow velocity was significantly and positively correlated with the daytime sap flow velocity (Table 5), and the fit was higher in the early growing season than that in the late growing season (early growing season: *R*^2^ = 0.51, *P* < 0.01; late growing season: *R*^2^ = 0.36, *P* < 0.01)(Figure 4). The fit between daytime and nighttime sap flow velocities was *R*^2^ = 0.52 for the entire study period. We can conclude that daytime and nighttime sap flow velocities of *F. pennsylvanica* are significantly and positively correlated. Table 5 indicates that a highly significant positive correlation exists between daytime and nighttime sap flow velocities with or without water stress, but the correlation is significantly higher without water stress. Therefore, an increase in daytime sap flow velocity will likely to produce a higher nighttime sap flow velocity under ideal moisture conditions. A highly significant positive correlation exists between daytime and nighttime sap flows for both sunny and cloudy conditions, but no correlation was observed for rainy day condition. Daytime and nighttime sap flow velocities on sunny days demonstrated the maximum correlation, with a fit of *R*^2^ = 0.797 (Table 5).

**TABLE 5:** Relationship between daytime and nighttime sap flow velocity under different soil moisture and weather conditions.

Different conditions		Daytime vs. nighttime correlation coefficients	Linear regression equation
Period	Early growing season	0.716**	*V*_*Night*_ = 0.135*V*_*Day*_ − 0.050, *R*^2^ = 0.513
	Late growing season	0.602**	*V*_*Night*_ = 0.113*V*_*Day*_ − 0.850, *R*^2^ = 0.363
Soil moisture	Water stress	0.568**	*V*_*Night*_ = 1.201*V*_*Day*_ − 0.002, *R*^2^ = 0.323
	No-water stress	0.725**	*V*_*Night*_ = 0.679*V*_*Day*_ − 0.001, *R*^2^ = 0.606
Weather	Sunny	0.893**	*V*_*Night*_ = 1.203*V*_*Day*_5.077, *R*^2^ = 0.797
	Cloudy	0.788**	*V*_*Night*_ = 0.242*V*_*Day*_ − 0.653, *R*^2^ = 0.621
	Rainy	-0.235	

**P < 0.01, *P < 0.05.

Comparing the overall environmental factors in the early and late growing seasons (Supplementary Table 3), it can be seen that rainfall is significantly higher in the late growing season than in the early growing season. RH and T_*a*_ are also higher in the later than in the early growing season. However, R_s_ and VPD are higher in the early growing season than in the late growing season. The sap flow velocity at each of the two periods was correlated with the corresponding simultaneous driving factors to investigate the relationship between daytime and nighttime sap flow velocities at different periods and driving factors further (Table 6). T_*a*_ was the factor with the maximum effect on sap flow velocity during both daytime and nighttime in the early growing season. Factors R_s_ and VPD presented the maximum influence on the respective daytime and nighttime sap flow velocities in the late growing season.

**TABLE 6:** Partial correlation coefficients between sap flow velocity and driving factors at different periods.

Period	Daytime					Nighttime			
	RH	T_a_	VPD	R_s_	VWC	RH	T_a_	VPD	VWC
Early growing season	−0.081*	0.600**	−0.183**	0.222**	−0.029	0.094*	0.224**	0.184**	0.061
Late growing season	−0.148*	0.393**	−0.114	0.454**	−0.021	0.109	0.305**	0.345**	0.041

**P < 0.01, *P < 0.05.

In addition, influencing factors interacted with one another with complex effects, and partial influencing factors are usually highly correlated with one another (Supplementary Tables 4–7). PCA is commonly used to detect and explain the underlying structure of various covariates. PCA reduces not only the dimensionality of the data set but also condenses the relevant data set into a few composite variables that retain the majority of information of original variables and thus appropriately reflect the combined information of influencing factors. Therefore, PCA was performed on the driving factors of sap flow, and the results are shown in Supplementary Table 8. Two principal components were extracted under each period of day and night in the principal component analysis. Among them, principal components of daytime and nighttime in the early growing season can explain 74.21 and 85.53% of the total data information, respectively. Meanwhile, principal components of daytime and nighttime in the late growing season can explain 78.75 and 90.80% of the total data information, respectively.

Expressions for the first (F_1_) and second (F_2_) principal components of R_s_ (X_1_), T_*a*_ (X_2_), RH (X_3_), VPD (X_4_), and VWC (X_5_) are listed in Supplementary Table 8. Regression equations were established with F_1_ and F_2_ as independent variables and standardized sap flow velocity data (F) as dependent variables for the corresponding periods. Expressions and equations for each period are presented in Supplementary Table 9. Regression models for driving factors and sap flow velocity were obtained by reverting the standardized variables to the original variables.

The early growing season model is expressed as follows:

$${\text{V}}_{\text{Day}}=\,1.53\times 10^{-3}\,{\text{x}}_{1}+4.68\,{\text{x}}_{2}-0.12\,{\text{x}}_{3}+0.78\,{\text{x}}_{4}+31.82\,{\text{x}}_{5}-32.76$$

$${\text{V}}_{\text{Night}}=\,1.49\,{\text{x}}_{2}-4.21\times 10^{-3}\,{\text{x}}_{3}+0.42\,{\text{x}}_{4}+8.21\,{\text{x}}_{5}-3.20$$

The late growing season model is expressed as follows:

$${\text{V}}_{\text{Day}}=\,7.34\times 10^{-3}\,{\text{x}}_{1}+4.14\,{\text{x}}_{2}-0.18\,{\text{x}}_{3}+0.79\,{\text{x}}_{4}+11.09\,{\text{x}}_{5}+7.06$$

$${\text{V}}_{\text{Night}}=2.72{\text{x}}_{2}+-0.10{\text{x}}_{3}+0.63{\text{x}}_{4}+4.93{\text{x}}_{5}-1.62$$

## Discussion

### Daytime and Nighttime Characteristics of Sap Flow Velocity and Influencing Factors

The sap flow velocity of *F. pennsylvanica* demonstrated an evident diurnal rhythm. The diurnal alternation of sap flow velocity exhibited a single peak with slight fluctuations. This finding is consistent with the results of Hua et al. (2021). The low and stable level of *F. pennsylvanica* sap flow velocity indicated that the plant root system still maintains a certain degree of water uptake capacity at night to meet the water balance and replenish the water loss caused by transpiration during the day (Han et al., 2019). Daytime sap flow velocity is significantly greater than the nighttime counterpart because of strong transpiration during the daytime. The xylem water column generates tension and a large amount of water enters the roots; the water enters the tree in a passive way. The sap flow at night was caused by root water potential, and water was actively absorbed into the tree to replace the water lost by daytime transpiration (Wu et al., 2020). In addition, the sap flow is more active in the first half of the night than that in the second half of the night. This phenomenon is mainly because the transpiration during the daytime causes a water potential difference between the root system and the soil of the tree after sunset. Thus, a high sap flow velocity occurs immediately after nightfall to replenish the water. The sap flow velocity decreases as the tree water deficit continues to decrease (Jie et al., 2019). Note that the sap flow velocity of *F. pennsylvanica* varied every month. The sap flow velocity was significantly higher in July, August, and September than that in April, May, and June during the study period. The sap flow velocity was highest in July but the lowest in April. Previous studies on the interannual variation of sap flow velocity also indicated that the maximum sap flow velocity occurs in July likely due the increased summer precipitation that provided satisfactory soil moisture conditions for plants (Iqbal et al., 2021). The nighttime sap flow velocity from June to September is likely due to high daytime water depletion that results in water deficit in trees. Hence, the nighttime sap flow velocity is kept high to maintain the water balance of trees taking up water at night to replace the water loss during the day (Han et al., 2019). VPD exerts a strong influence on the sap flow velocity in the Pearson’s correlation analysis (Supplementary Tables 2, 10, 11); however, the results of the partial correlation analysis after removing the correlation between environmental factor variables indicated that the degree of influence of VPD on sap flow decreases significantly (Tables 2, 4, 6). The positive effect of VPD on sap flow became negative after removing the correlation between environmental factors even during the daytime. This particular phenomenon was also seen in the study by Antezana-Vera and Marenco (2021). The effect of net VPD on the sap flow is negative during the daytime and positive during nighttime when the influence of other factors on VPD is removed. However, the effect of VPD on daytime sap flow became positive when effects of other environmental factors on VPD were considered. Thus, assuming that net VPD restrained daytime sap flow velocity and the influence of T_a_, R_s_, RH on VPD results in the positive effect of VPD on the sap flow while considering the influence of T_a_ and R_s_ and RH on VPD is reasonable because VPD is a function of T_a_ and RH and T_a_ is also influenced by R_s_ (Antezana-Vera and Marenco, 2020).

### Response of Sap Flow Velocity to Microclimatic Factors Under Different Water Conditions

The main factors influencing the variation of sap flow velocity include physiological structure, soil water supply conditions, and meteorological factors. Among them, soil water affects the overall level of sap flow, and meteorological factors influence transient changes in sap flow (Granier et al., 2000a). Soil moisture content is a key factor affecting the overall level of sap flow velocity. Rainfall can provide temporal recharge of soil water that can lead to increased sap flow velocity through hydraulic conduction in the xylem (Shen et al., 2015). The degree of response of sap flow velocity to its associated factors varies under different water conditions. Previous studies on the relationship between sap flow and environmental factors under two different soil conditions indicated that diverse water supply conditions are dominant factors leading to various responses of sap flow to meteorological factors (Wu et al., 2018). In addition, the sap flow is susceptible to the influence of meteorological factors when the soil water content is sufficient (Chen et al., 2014a). Meteorological factors interact with one another, and changes in the sap flow are the result of a combination of meteorological factors. Therefore, the sensitivity of sap flow to the overall meteorological factors should be considered a composite result without excluding the correlation between factors. The results of Pearson as correlation analysis are listed in Supplementary Table 2. The sensitivity of sap flow velocity to meteorological factors was greater without water stress during the daytime but higher under water stress at night. Another study has also pointed out that moso bamboo produces considerable nighttime sap flow under drought conditions (Tong et al., 2021). The increasing nighttime sap flow can reduce the formation of xylem embolism and air pockets in plants and avoid hydraulic failure due to drought while alleviating their water deficit (Klein et al., 2018).

### Relationship Between Daytime and Nighttime Sap Flow Velocity

Daytime sap flow velocity exerted a highly significant positive effect (*P* < 0.01) on nighttime sap flow velocity throughout the study period, and the correlation was strong in the early part of the growing season. Zhao et al. (2017) indicated that this phenomenon might be due to many rainfall events in the late growing season. The results of our study indicated that no significant correlation exists between daytime and nighttime sap flows on rainy days. The number of rainy days in the late growing season accounted for one-half of the total number of days (23 days in July, 10 days in August, and 13 days in September). Therefore, we assumed that the lower correlation between daytime and nighttime in the late growing season compared to the early growing season is caused by the high number of rainy days in the late growing season. In addition, a highly significant positive correlation is observed between daytime and nighttime sap flow velocities both with and without water stress periods, although the correlation was significantly higher in the absence of water stress. Therefore, an increase in daytime sap flow velocity will likely produce high nighttime sap flow velocity under ideal moisture conditions. Oogathoo et al. (2020) indicated that saturated VPD and R_s_ are the main drivers of transpiration. The results of our study indicated that daytime sap flow is correlated with T_a_ and R_*s*,_ and nighttime sap flow is correlated with T_a_ and VPD, except in the case of water stress. Therefore, the sap flow of *F. pennsylvanica* is mainly controlled by hydrothermal factors. The correlation between plant nighttime sap flow and VPD can be used to determine whether nighttime sap flow is used for transpiration (Dawson et al., 2007). The presence of transpiration by nighttime sap flow of *F. pennsylvanica* can be assumed because a highly significant correlation between nighttime sap flow velocity and VPD was revealed in this study. Hence, we can speculate that the highly significant positive correlation between nighttime and daytime sap flow velocities will likely lead to rehydration of *F. pennsylvanica* at night. The trees suffered from severe water deficit in the evening due to the strong transpiration and high sap flow during the day. High nighttime sap flow was generated to replenish tree water. These findings clearly demonstrated that the nighttime sap flow of *F. pennsylvanica* during the study period is a combination of nocturnal rehydration and transpiration.

## Conclusion

During the study period, the sap flow velocity was highest in July and lowest in April. The daytime sap flow velocity was correlated with T_a_ and R_s_ in all months, and the nighttime sap flow velocity was correlated with T_a_ and VPD in all months in terms of influencing factors. Therefore, the sap flow of *F. pennsylvanica* is mainly controlled by hydrothermal factors. A particular finding of this paper is that the effect of net VPD on daytime sap flow is negative, but the effect of VPD on sap flow when combined with other environmental factors is positive. Therefore, when considering the role of VPD, it is important to analyze it in different situations. This result furthered our knowledge of the relationship between plants and environmental factors.

Higher nighttime sap flow velocity is produced under water stress conditions. Therefore, more emphasis should be placed on the nocturnal water consumption of plants in arid areas than in wet areas. Daytime and nighttime sap flow velocities of *F. pennsylvanica* were highly significantly and positively correlated. A highly significant positive correlation exists between daytime and nighttime sap flows for both sunny and cloudy conditions. Therefore, the nighttime sap flow can be well predicted by the daytime flow in the absence of rainy weather conditions. The principal components of driving factors were extracted using principal component regression analysis, and a regression model of driving factors and the sap flow velocity was constructed. It provides a theoretical basis for the prediction of sap flow velocity and the management of *F. pennsylvanica*.

Issues related to trunk sap flow still require further exploration. Monitoring may be ineffective at extremely low sap flow and reverse sap flow cannot be observed due to limitations of the TDP method. Therefore, the heat ratio method (HRM) is considered in our follow-up investigation because it can measure zero, low, and reverse flows of plants (Sun et al., 2022). Various forms of stomatal regulation at different times due to the circadian rhythm of plants affect transpiration (Cirelli et al., 2015). The regulation of stomatal conductance through the starch metabolism of the plant (Lascève et al., 2010) presents a corresponding effect on transpiration. The combination of environmental factors and physiological effects requires further investigation to determine whether environmental factors can affect plant physiological processes and their corresponding responses.

## Data Availability Statement

The raw data supporting the conclusions of this article will be made available by the authors, without undue reservation.

## Author Contributions

YS drafted, revised the manuscript, and contributed to data analysis. XW and YqS participated in the experiments and performed the maintenance of the instruments. HW designed, coordinated the study, and revised the manuscript. All authors contributed to the article and approved the submission.

## Conflict of Interest

The authors declare that the research was conducted in the absence of any commercial or financial relationships that could be construed as a potential conflict of interest.

## Publisher’s Note

All claims expressed in this article are solely those of the authors and do not necessarily represent those of their affiliated organizations, or those of the publisher, the editors and the reviewers. Any product that may be evaluated in this article, or claim that may be made by its manufacturer, is not guaranteed or endorsed by the publisher.
