# Supplementary material for: Sap Flow Velocity in Fraxinus pennsylvanica in Response to Water Stress and Microclimatic Variables
Source: Front Plant Sci. 2022 May 10;13:884526. doi: 10.3389/fpls.2022.884526 (PMC9127660; doi:10.3389/fpls.2022.884526)
Supplement: Supplementary file 1 [file Data_Sheet_1.PDF]

Supplementary Material

Table 1 Soil moisture conditions and precipitation in site by month

Month	VWC($cm^3 \cdot cm^{-3}$)				REW	Precipitation(mm)
	5cm	20cm	30cm	40cm	5-40cm	
April	0.23±0.02	0.27±0.01	0.29±0.01	0.32±0.01	0.25±0.02	9.40
May	0.15±0.01	0.31±0.01	0.31±0.01	0.33±0.01	0.22±0.02	8.64
June	0.20±0.04	0.33±0.03	0.34±0.03	0.34±0.03	0.43±0.13	55.88
July	0.24±0.07	0.32±0.05	0.32±0.05	0.37±0.04	0.47±0.12	156.21
August	0.21±0.04	0.40±0.03	0.29±0.03	0.35±0.02	0.28±0.13	45.72
September	0.20±0.05	0.25±0.04	0.27±0.04	0.32±0.03	0.17±0.15	56.39

Table 2 Pearson coefficients between sap flow velocity and meteorological factors under different water conditions

Water conditions	Daytime				Nighttime		
	RH	T_a	VPD	R_s	RH	T_a	VPD
Water stress	-0.590**	0.571**	0.579**	0.641**	-0.738**	0.458**	0.696**
No-water stress	-0.784**	0.812**	0.781**	0.672**	-0.603**	0.111**	0.613**

** P<0.01, * P<0.05.

Table 3 Environmental factors condition at different period

Factors	Early growing season		Late growing season	
	Daytime	Nighttime	Daytime	Nighttime
RH (%)	48.62±25.89	60.79±23.19	65.61±19.52	83.18±12.42
T_a (℃)	21.72±6.26	18.32±5.29	26.51±3.07	23.25±2.26
VPD (kPa)	1.47±1.02	0.83±0.54	1.30±0.81	0.51±0.40
VWC ($cm^3 \cdot cm^{-3}$)	0.32±0.02	0.32±0.02	0.33±0.03	0.33±0.03
R_s ($MJ \cdot m^{-2} \cdot d^{-1}$)	11.25±4.97		7.78±3.22	
Total precipitation (mm)	73.92		258.32	

Table 4 Correlation analysis between the various environmental factors during early growing season daytime

Early growing season daytime	R_s	RH	T_a	VPD	VWC
R_s	1	-0.394**	0.014	0.203**	-0.161**
RH		1	-0.132**	-0.755**	0.409**
T_a			1	0.675**	0.275**
VPD				1	-0.170**
VWC					1

** P<0.01, * P<0.05.

Table 5 Correlation analysis between the various environmental factors during late growing season nighttime

Late growing season nighttime	RH	T_a	VPD	VWC
RH	1	-0.532**	-0.894**	0.367**
T_a		1	0.584**	0.091
VPD			1	-0.195**
VWC				1

** P<0.01, * P<0.05.

Table 6 Correlation analysis between the environmental factors during late growing season daytime

Late growing season daytime	R_s	RH	T_a	VPD	VWC
R_s	1	-0.177**	0.246**	0.168**	0.017
RH		1	-0.750**	-0.983**	0.367**
T_a			1	0.827**	0.001
VPD				1	-0.283**
VWC					1

** P<0.01, * P<0.05.

Table 7 Correlation analysis between the various environmental factors during early growing season nighttime

Early growing season nighttime	RH	T_a	VPD	VWC
RH	1	0.276**	-0.771**	0.446**
T_a		1	0.328**	0.378**
VPD			1	-0.243**
VWC				1

** P<0.01, * P<0.05.

Table 8 Principal component analysis of the driving factors of sap flow velocity at different periods

Item	Early growing season				Late growing season			
	Daytime		Nighttime		Daytime		Nighttime	
	PC1	PC2	PC1	PC2	PC1	PC2	PC1	PC2
Eigenvalue	2.258	1.452	2.034	1.387	2.851	1.087	2.546	1.086
Variance Contribution	45.167	29.042	50.853	34.677	57.018	21.734	63.647	27.153
Cumulative contribution rate	45.167	74.209	50.853	85.529	57.018	78.752	63.647	90.801
R_s	0.477	-0.351			0.294	0.616		
T_a	0.518	0.788	0.250	0.907	0.869	0.281	0.711	0.560
RH	-0.878	0.272	0.944	-0.055	-0.967	0.121	-0.961	0.088
VPD	0.930	0.285	-0.784	0.607	0.980	-0.045	0.983	0.025
VWC	-0.358	0.744	0.682	0.440	-0.338	0.782	-0.387	0.874

Table 9 Expression of principal components of the driving factors

Period	Daytime	Nighttime
Early growing season	$F_1 = 0.32Z_1 + 0.34Z_2 - 0.58Z_3 + 0.62Z_4 - 0.24Z_5$	$F_1 = 0.18Z_2 + 0.66Z_3 - 0.55Z_4 + 0.48Z_5$
	$F_2 =- 0.29Z_1 + 0.65Z_2 + 0.23Z_3 + 0.24Z_4 + 0.62Z_5$	$F_2 = 0.77Z_2 - 0.05Z_3 + 0.52Z_4 + 0.37Z_5$
	$F = 0.07Z_1 + 0.41Z_2 - 0.26Z_3 + 0.28Z_4 - 0.25Z_5$	$F = 0.43Z_2 - 0.03Z_3 + 0.29Z_4 + 0.21Z_5$
Late growing season	$F_1 = 0.17Z_1 + 0.51Z_2 - 0.57Z_3 + 0.58Z_4 - 0.20Z_5$	$F_1 = 0.45Z_2 - 0.60Z_3 + 0.62Z_4 - 0.24Z_5$
	$F_2 = 0.59Z_1 + 0.27Z_2 + 0.12Z_3 - 0.04Z_4 + 0.75Z_5$	$F_2 = 0.54Z_2 + 0.08Z_3 + 0.02Z_4 + 0.84Z_5$
	$F = 0.24Z_1 + 0.29Z_2 - 0.20Z_3 + 0.23Z_4 - 0.14Z_5$	$F = 0.31Z_2 - 0.20Z_3 + 0.23Z_4 + 0.14Z_5$

Table 10 Pearson correlation coefficients between sap flow velocity and meteorological factors for each month

Month	RH		T_a		VPD		R_s
	Daytime	Nighttime	Daytime	Nighttime	Daytime	Nighttime	Daytime
4	-0.371**	-0.236**	0.633**	0.282**	0.544**	0.346**	0.277**
5	-0.441**	-0.491**	0.399**	0.468**	0.439**	0.637**	0.459**
6	-0.558**	-0.551**	0.597**	0.583**	0.517**	0.611**	0.432**
7	-0.731**	-0.325**	0.851**	0.567**	0.752**	0.381**	0.508**
8	-0.751**	-0.508**	0.781**	0.408**	0.747**	0.562**	0.476**
9	-0.745**	-0.924**	0.751**	0.746**	0.701**	0.707**	0.504**

** $P<0.01$, * $P<0.05$.

Table 11 Pearson correlation coefficients between sap flow velocity and driving factors at different periods

Period	Daytime					Nighttime			
	RH	T_a	VPD	R_s	VWC	RH	T_a	VPD	VWC
Early growing season	-0.190**	0.694**	0.515**	0.208**	0.374**	0.016	0.637**	0.406**	0.203**
Late growing season	-0.617**	0.709**	0.638**	0.497**	-0.089	-0.526**	0.575**	0.581**	0.011

** $P<0.01$, * $P<0.05$.
